# The Effect of β_2_-Adrenoceptor Agonists on Leucocyte-Endothelial Adhesion in a Rodent Model of Laparotomy and Endotoxemia

**DOI:** 10.3389/fimmu.2020.01001

**Published:** 2020-05-21

**Authors:** Mansoor Nawaz Bangash, Tom E. F. Abbott, Nimesh S. A. Patel, Charles Johnston Hinds, Christoph Thiemermann, Rupert Mark Pearse

**Affiliations:** ^1^Department of Critical Care & Anaesthesia, University Hospitals Birmingham NHS Trust, Birmingham, United Kingdom; ^2^Centre for Translational Medicine & Therapeutics, William Harvey Research Institute, Queen Mary University of London, London, United Kingdom

**Keywords:** adrenergic β_2_ receptor agonists, dopexamine, inflammation, microcirculation, surgery

## Abstract

**Background:** The β_2_-adrenoceptor agonist dopexamine may possess anti-inflammatory actions which could reduce organ injury during endotoxemia and laparotomy. Related effects on leucocyte-endothelial adhesion remain unclear.

**Methods:** Thirty anesthetized Wistar rats underwent laparotomy followed by induction of endotoxemia with lipopolysaccharide and peptidoglycan (*n* = 24) or sham (*n* = 6). Animals received dopexamine at 0.5 or 1 μg kg^−1^ min^−1^ (D0.5 and D1), salbutamol at 0.1 μg kg^−1^ min^−1^, or saline vehicle (Sham and Control) for 5 h. Intravital microscopy was performed in the ileum of the small intestine to assess leucocyteendothelial adhesion, arteriolar diameter, and functional capillary density. Global hemodynamics and biochemical indices of renal and hepatic function were also measured.

**Results:** Endotoxemia was associated with an increase in adherent leucocytes in post-capillary venules, intestinal arteriolar vasoconstriction as well-reduced arterial pressure and relative cardiac index, but functional capillary density in the muscularis was not significantly altered. Dopexamine and salbutamol administration were associated with reduced leucocyte-endothelial adhesion in post-capillary venules compared to control animals. Arteriolar diameter, arterial pressure and relative cardiac index all remained similar between treated animals and controls. Functional capillary density was similar for all groups. Control group creatinine was significantly increased compared to sham and higher dose dopexamine.

**Conclusions:** In a rodent model of laparotomy and endotoxemia, β_2_-agonists were associated with reduced leucocyte-endothelial adhesion in post-capillary venules. This effect may explain some of the anti-inflammatory actions of these agents.

## Introduction

Complications following major gastrointestinal surgery have a significant impact on both short and long-term survival (1–3). Inotropic agents may have important effects on outcomes for this patient group (4). Dopexamine is a dopamine analog with agonist activity at β_2_-adrenoceptor and dopaminergic receptors. This spectrum of activity confers vasodilator actions in addition to chronotropic and mild inotropic effects (5). Dopexamine has been used to increase cardiac output and hence tissue oxygen delivery in several trials of peri-operative haemodynamic therapy (6, 7). Other cardiovascular effects of dopexamine may include improved tissue microvascular flow and oxygenation (8). Various groups have studied the effects of dopexamine in patients following major gastrointestinal surgery (6, 9), with promising results, although the findings of a recent large trial were inconclusive (7).

Investigators have previously demonstrated potent anti-inflammatory effects of β_2_-adrenoceptor agonists (10–18), in particular dopexamine (19–22). However, it is unclear that β_2_-adrenoceptor agonism is responsible for the dopexamine-dependent reduction of leucocyte-endothelial adhesion seen in several endotoxemia studies (19, 20). The findings of previous laboratory and clinical investigations suggest dopexamine may improve tissue microvascular flow and oxygenation (8, 19, 20, 23–26), and it is thought that these effects may account for much of the potential benefit of inotropic agents in the critically ill (27). However, in a previous laboratory study from our group, the haemodynamic actions of dopexamine infusion appeared to be less important than anti-inflammatory effects, including decreased plasma cytokine levels, modulation of neutrophil CD11b surface expression, and decreased pulmonary neutrophil infiltration (28). We sought to further clarify how leucocyte-endothelial adhesion under endotoxemia might relate to the β_2_-adrenoceptor agonist effects of dopexamine, and its effects on arterial pressure, cardiac output and the microcirculation.

We therefore investigated the effects of dopexamine on leucocyte-endothelial adhesion within the microcirculation. Our hypothesis was that, in a rodent model of laparotomy and endotoxemia, dopexamine would decrease leucocyte-endothelial adhesion in intestinal post-capillary venules, through β_2_-adrenoceptor mediated actions. The relative contribution of β_2_-adrenoceptor agonism to these effects was assessed by using the β_2_ adrenergic agonist salbutamol as a comparator.

## Materials and Methods

Thirty male Wistar rats (240–340 g) received a standard diet and water *ad libitum* before the experiments. All procedures were performed with institutional approval and in accordance with the United Kingdom Home Office Guidance on the Operation of the Animals (Scientific Procedures) Act 1986 under the project license PPL 70/6526. Anesthesia was induced by intra-peritoneal injection of thiopental (120 mg kg^−1^) and maintained with supplementary injections administered according to regular testing for limb withdrawal to a standard stimulus. Animals were placed on a warming mat to maintain a core temperature of 37 ± 0.5°C. A tracheostomy was performed, following which a short section of polyethylene tubing (internal diameter, 1.67 mm) was inserted to maintain airway patency and to facilitate spontaneous respiration. The right carotid artery was cannulated to allow blood sampling and continuous monitoring of heart rate (HR) and mean arterial pressure (MAP). The left jugular vein was cannulated for drug and fluid administration.

A 2 cm midline incision was then made through the abdominal wall to expose the peritoneum. Following laparotomy, bowel was evacuated into a moist cotton receptacle. Blunt dissection was then performed to access the abdominal vasculature. After isolation from the vena cava, a 1.5 mm ultrasonic aortic transit time flow probe (MA1.5PRB; Transonic Systems Inc., Ithaca, USA) was placed on the infra-renal aorta to measure aortic blood flow allowing calculation of relative stroke volume and relative cardiac index. The bowel was then replaced in the abdominal cavity, except for a loop of ileum just proximal to the caecum. The exposed bowel was kept moist by the application of 0.9% saline drops through a pipette. The laparotomy incision above and below the exit of the terminal ileal loop from the abdomen was then closed with 5.0 vicryl to prevent excessive insensible fluid losses. The animal was maintained on a warming mat on an intravital microscopy platform and placed in the right lateral position so the ileal loop fell on to a raised section of the platform at the level of the laparotomy incision. The temperature of the raised section was thermostatically controlled at 37.5°C to ensure the temperature of the exposed bowel was similar to the core temperature. This position did not interfere with the ability of the ultrasonic probes to measure aortic blood flow. Subsequently the bowel was covered with Saran wrap to prevent evaporative losses from its surface and maintain bowel microvascular integrity (29). This was followed by a 5 ml kg^−1^ bolus of normal saline to replace insensible fluid losses and a 15 min stabilization period to allow microvascular flow to stabilize. A first set of arterial blood samples was then taken (see below), the volume being replaced with an equal volume of normal saline. Animals were allowed to stabilize for 15 min before being allocated randomly to one of five groups (sham, control, D0.5, D1, S).

Endotoxemia was induced over a 10 min period in four of five groups by administering 1 ml kg^−1^ of a solution containing Escherichia coli lipopolysaccharide 0111:B4 (LPS, 1 mg ml^−1^) and peptidoglycan (PepG, 0.3 mg ml^−1^) intravenously, the sham group received 0.9% saline vehicle alone. In all groups this was followed by an infusion of 0.9% saline at 4.3 ml kg^−1^ h^−1^ though different doses of dopexamine or salbutamol were added to the D0.5, D1, and S groups' infusion fluid. This resulted in dopexamine infusion rates of 0.5 and 1 μg kg^−1^ min^−1^ for groups D0.5 and D1, respectively, and a salbutamol infusion rate of 0.1 μg kg^−1^ min^−1^ in group S. This dose of salbutamol was selected as previous studies conducted in isolated guinea–pig tracheal preparations showed a 10-fold greater potency of salbutamol at the β_2_-adrenoceptor when compared with dopexamine (30). Intravital microscopy in the intestinal ileum was performed after 150 min, midway through resuscitation. It was not possible to measure global hemodynamics during this procedure. The experiment ended after 5 h of resuscitation when the heart and lungs were excised.

### Analysis of Plasma Lactate, Base Deficit, and Renal and Hepatic Function

Two hundred microliter of blood was taken at baseline and at the end of the experiment for measurement of plasma lactate concentration (Accutrend Lactate; Roche Diagnostics, Basel, Switzerland) and base deficit (Radiometer ABL77, Copenhagen, Denmark). A 1ml blood sample was also taken at the end of the experiment for measurements of urea, creatinine, alanine aminotransferase, and aspartate aminotransferase by a commercial veterinary laboratory (IDEXX Laboratories Ltd, Sussex, UK).

## Measurement of Aortic Blood Flow

A 1.5 mm perivascular probe was applied with water-soluble sonicating gel and sited as described earlier. The probe was connected to a TS420 monitor (Transonic Systems Inc., Ithaca, USA), which was connected to a Powerlab/8SP monitoring system (AD Instruments). This allowed continuous recording of aortic blood flow and HR, and calculation of relative stroke volume and relative cardiac output (relative as infra-renal aortic blood flow is not equivalent to cardiac output). Aortic blood flow was indexed to body weight to provide a measure of changes in relative stroke volume index (SVI) and relative cardiac index (CI). Probe calibration was performed daily according to the manufacturer's instructions before experiments.

### Intravital Microscopy (IVM)

Fifteen minutes before the midpoint of fluid resuscitation, 0.2 ml of 0.17 g L^−1^ rhodamine 6G (Sigma-Aldrich, Gillingham, UK) was administered intravenously to enhance the visibility of leucocytes. The animal platform was then transferred to the stage of an intravital microscope. Fluorescence microscopy was carried out using an Olympus BX61W1 microscope (Carl Zeiss Ltd.) connected to an Olympus BXUCB lamp, Uniblitz VCMD1 shutter driver and DG4-700 shutter instrument. Recordings were captured using Slidebook 5.0 software (Intelligent Imaging TTL) and saved for later offline analysis. All images were taken at x40 magnification. Leucocyte rolling and adhesion (>30 s stationary) was quantified in ileal post-capillary venules: the course of microvessels of the ileal submucosal layer was followed from collecting venules (V1) to postcapillary venules (V3), the latter being selected for analysis. Vessel length and diameter was measured and recorded. Images were recorded for a minimum of 40 s. A further 0.2 ml of 250 mg kg^−1^ ml^−1^ of FITC labeled bovine albumin (Sigma-Aldrich) was then administered intravenously in order to measure functional capillary density (FCD) and arteriolar diameters: the course of microvessels of the ileal submucosal layer was followed from supply arterioles (A1) to pre-capillary arterioles (A3), the latter being selected for analysis. Vessel diameter was measured and recorded. Capillaries were identified in the circular and longitudinal layers of the ileum and images were recorded for a minimum of 40 s. These images were later analyzed offline. The platform was then removed from the microscope stage and observations continued as before.

Recordings of intravital videos were stored electronically. These files were later analyzed offline using Slidebook 5.0 Reader [Intelligent Imaging Innovations (3i)] by an observer blinded to the experiment groups. Leucocyte adhesion was quantified and indexed to endothelial surface area (mm^2^), calculated from the diameters and lengths of the vessel segments studied and assuming cylindrical vessel geometry. Firmly adherent leucocytes were defined as those that did not move or detach from the endothelial lining within an observation period of 30 s. FCD was calculated as the total length of perfused capillaries indexed to the visualized area (mm^−1^).

### Statistical Analysis

Data were presented as Mean (SEM) unless expressed otherwise and specifically. Kolmogorov-Smirnov normality testing was performed for all groups. Normally distributed data were tested using one-way analysis of variance (ANOVA) for comparison across all groups at a given time point. Post-testing was performed with Bonferroni's tests. Occasionally when ANOVA revealed significant results but post-tests did not indicate which group was responsible, *t*-tests (with or without Welch's correction depending on the variance of data) were performed to gain additional insight to the data. When data were not normally distributed in at least one group for any measurement, data were expressed as median (IQR) and the Kruskal-Wallis test was used in place of one-way ANOVA with Mann Whitney post-tests (and a Bonferroni correction). Two-tailed paired *t*-tests were used to compare hemodynamics at baseline with those at other time points for animals within the same group. Data were analyzed with PrismGraph 4.0 (GraphPad Software, San Diego, USA). Significance was set at *p* < 0.05.

## Results

**Figure 1:**
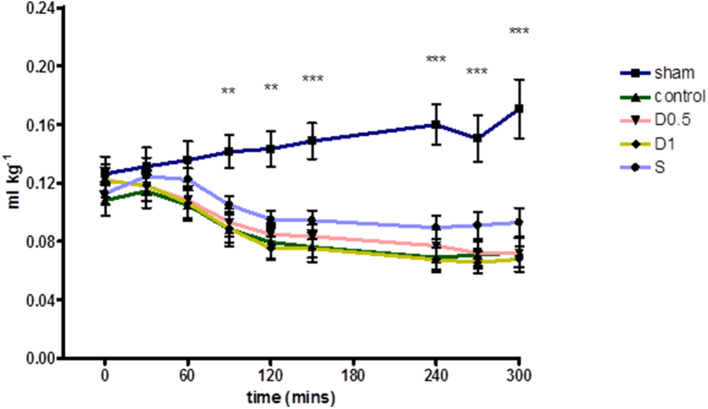
Relative Stroke Volume Index during 5 h of laparotomy and endotoxemia recorded every 30 min. Values not plotted for t180–t210 [animals were undergoing IVM at this time and aortic flow and HR could not be measured (therefore relative SVI could not be calculated)]. Relative stroke volume index in controls differed significantly for most of the experiment and until its end when compared to shams. However, no significant differences in relative stroke volume index were observed between controls and groups treated with dopexamine or salbutamol at any time. The mean change in relative stroke volume index from baseline to end experiment was also significant in all groups except salbutamol treated animals (also see Table 1). Data presented as mean (SEM). One-way ANOVA at each time point (Bonferroni's post-tests, **p < 0.01, ***p < 0.001 vs. controls).

**Figure 2:**
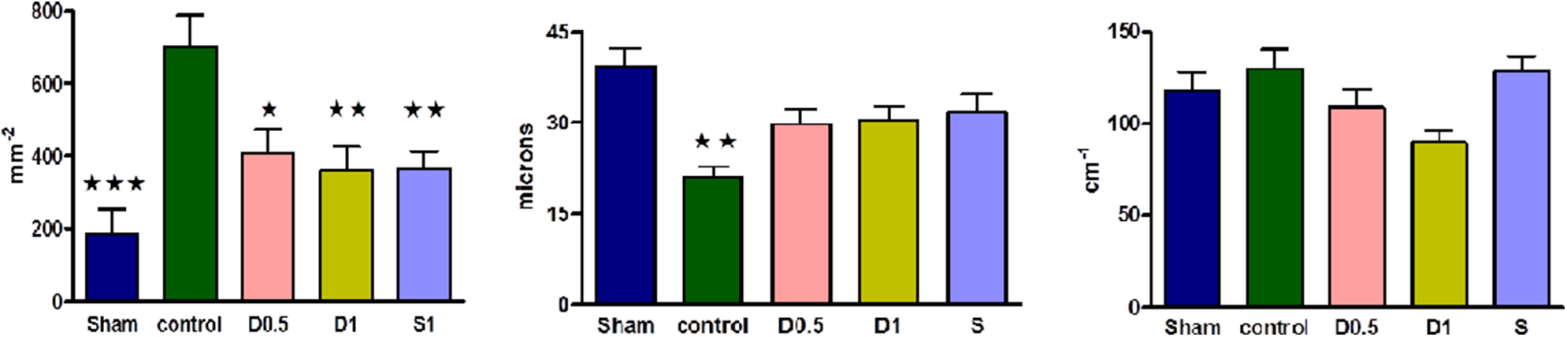
(Left) Numbers of adherent leucocytes per square mm of endothelium in ileal post-capillary venules at 2.5 h of laparotomy and endotoxemia (*n* = 6 all groups). Numbers of vessels observed per group ranged from 8 to 18. Sham, dopexamine, and salbutamol treated groups demonstrated significantly less adhesion than controls. Data presented as mean (SEM). One-way ANOVA (Bonferroni's post-tests, *p < 0.05, **p < 0.01, ***p < 0.001 vs. controls). (Mid) Intestinal arteriolar diameters of the ileum at 2.5 h of laparotomy and endotoxemia (*n* = 6 all groups). Numbers of vessels measured per group ranged from 8 to 19. When compared to shams and unlike controls, ileal arteriolar diameters were not significantly reduced in dopexamine and salbutamol treated groups. Data presented as mean (SEM). One-way ANOVA (Bonferroni's post-tests, **p < 0.01 vs. shams). (Right) Intestinal functional capillary density in longitudinal layers of the ileal muscularis at 2.5 h of laparotomy and endotoxemia (*n* = 6 all groups). Number of images per group ranged from 16 to 25. Groups were significantly different with respect to longitudinal FCD at 2.5 h. Data presented as mean (SEM). One-way ANOVA *p* = 0.024 (no groups positive in post-tests, although *p* < 0.01 when comparing control and D1 group with unpaired *t*-test with Welch's correction).

**Figure 3:**
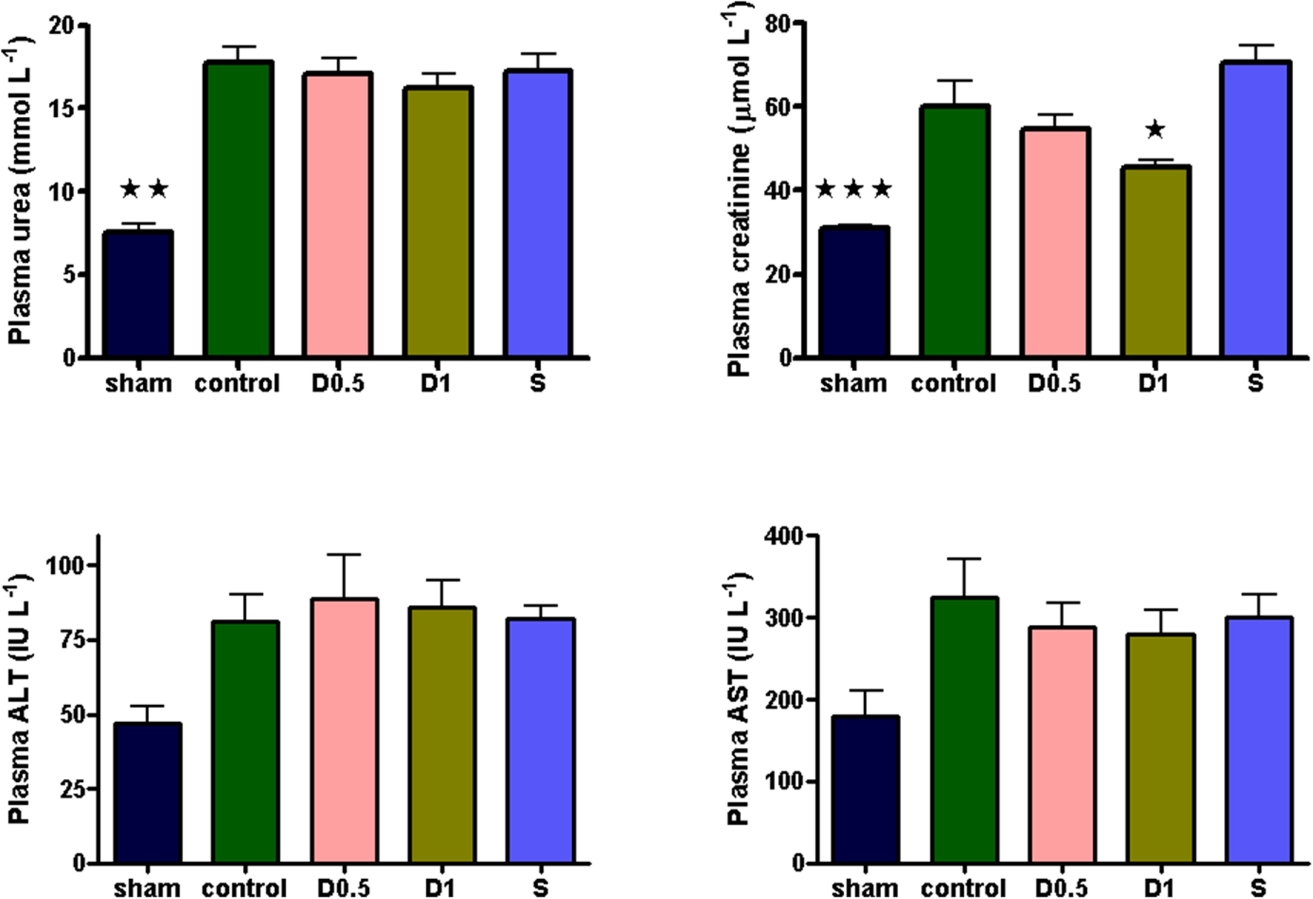
Plasma urea, creatinine, ALT and AST sampled 5 h after laparotomy and endotoxemia (*n* = 6 all groups). 5 h of laparotomy and endotoxemia caused significant acute kidney injury in controls. The degree of injury was significantly less in the D1 group. All data presented as mean (SEM). Plasma urea and creatinine: One-way ANOVA (Bonferroni's post-tests, *p < 0.05, **p < 0.01, ***p < 0.001 vs. controls). ALT: One-way ANOVA, *p* = 0.0246 (no groups positive in post-tests).

Baseline characteristics and fluid management are described in (Table 1 and Supplementary Table 1). There were no significant differences between groups regarding weight or volume of fluid received. Animals in the sham group required a slightly greater dose of thiopental to maintain anesthesia (Supplementary Table 1). There were no baseline differences in hemodynamics, base deficit, lactate or hematocrit. In the sham group, MAP and HR did not change significantly but CI and SVI increased progressively (Table 1, Figure 1, Supplementary Table 1, Supplementary Figures 1, 2). Compared with the sham group and baseline, controls had a significantly higher HR (*p* < 0.05) and a significantly lower SVI and CI at 5 h (Table 1, Figure 1, Supplementary Figures 1, 2). At this point control group plasma base deficit and lactate were increased compared with sham animals, the latter significantly (*p* < 0.05) (Table 1, Supplementary Figure 3). Compared to shams, there were more firmly adherent leucocytes (control: 703 ± 86 mm^−2^ vs. sham: 186 ± 68 mm^−2^; *p* < 0.001), and fewer rolling leucocytes in the post-capillary venules of control animals (Figure 2, Supplementary Figure 4). Intestinal arteriolar diameters were reduced in control animals [control: 21 ± 2 μm vs. sham: 39 ± 3 μm; *p* < 0.01; Figure 2 (mid)] although FCD in the muscularis and its component circular and longitudinal layers did not differ significantly from shams [Figure 2 (right), Supplementary Figure 4]. Endotoxemia was associated with acute kidney injury but not liver dysfunction (Figure 3).

**Table 1:** Fluid administered, temporal changes in blood gas and haemodynamic parameters for each group (*n* = 6 all groups).

	**Sham**	**Control**	**D 0.5**	**D1**	**S**
Administered fluid (ml kg^−1^)	29.9 (29.2–30.5)	29.4 (29.3–29.5)	29.8 (29.4–30.5)	30.1 (29.4–30.5)	29.8 (29.4–30.4)
Baseline HR (bpm)	378 (357–421)	399 (379–417)	415 (390–420)	379 (356–441)	392 (370–423)
Baseline MAP (mmHg)	120 (7)	111 (3)	114 (5)	108 (3)	110 (6)
End experiment HR (bpm)	371 (8)^***^	447 (12)	465 (15)	478 (9)	445 (5)
End experiment MAP (mmHg)	114 (95–133)	93 (69–101)	81 (76–106)	94 (79–106)	78 (70–106)
End experiment lactate (mmol L^−1^)	1.7 (0.2)^*^	3.4 (0.5)	3.1 (0.3)	2.6 (0.4)	3.8 (0.3)
End experiment base deficit (mmol L^−1^)	−0.6 (1.0)	4.5 (1.6)	2.9 (1.5)	3.3 (1.5)	6.0 (1.0)
Mean change in SVI during experiment (ml kg^−1^)	0.044 (0.014)^*^	−0.036 (0.008)^**^	−0.050 (0.009)^**^	−0.054 (0.003)^***^	−0.020 (0.011)
Mean change in CI during experiment (ml min^−1^ kg^−1^)	15.3 (6.2)	−10.5 (3.2)^*^	−16.1 (4.2)^*^	−14.4 (2.0)^***^	3.0 (4.9)

*There were no significant differences between groups in baseline hemodynamics or volumes of fluid administered. Over the course of the experiment in shams SVI significantly increased and CI also tended to increase, though HR and MAP did not change. In contrast HR significantly increased, while SVI and CI significantly decreased in control and dopexamine groups. MAP was significantly decreased, though this was not a consistent finding. HR also significantly increased in group S and although CI and SVI remained relatively stable MAP decreased significantly. A significantly lower lactate was seen in shams compared to controls. Data presented as mean (SEM) when all groups normally distributed, otherwise median (IQR) if ≥ 1 group not normally distributed. One-way ANOVA (post hoc Bonferroni's test*,

* *p < 0.05*,

** *p < 0.01*,

*** p < 0.001 vs. controls). Paired t-tests of baseline vs. end experiment for mean changes (^*^p < 0.05, ^**^p < 0.01, ^***^p < 0.001).

Dopexamine infusion had no significant effect on any haemodynamic parameters when compared to controls, except for an increase in heart rate (Table 1, Figure 1, Supplementary Table 1, Supplementary Figures 1, 2). Similarly, dopexamine was not associated with any improvement in plasma lactate or base deficit in endotoxaemic animals (Table 1, Supplementary Figure 3). The major finding of this study was that at the mid-point of resuscitation dopexamine significantly reduced leucocyte adhesion in post-capillary venules when compared to controls (D0.5: 409 ± 65 mm^−2^, *p* < 0.05 vs. control; D1: 361 ± 66 mm^−2^, *p* < 0.01 vs. control) (Figure 2). Furthermore, dopexamine prevented the reduction in arteriolar diameter observed in control animals [Figure 2 (mid)]. The effects of dopexamine on FCD were complex. There were significant differences between groups in longitudinal muscle FCD (one-way ANOVA *p* = 0.024) [Figure 2 (right)], but post-tests did not show which group was responsible for this difference. However, isolated *t*-tests comparing each group against controls reveal that only the D1 group had a significantly reduced longitudinal FCD compared to controls (unpaired *t*-test with Welch's correction, *p* = 0.0034). Dopexamine had no effect on FCD in the circular layer of the muscularis at any dose. When comparing total muscularis FCD for all groups, differences fell outside of the limits of statistical significance (one way ANOVA *p* = 0.058) (Supplementary Figure 4). Regarding organ function, renal function was improved in the D1 group compared to controls, but not the D0.5 animals (Figure 3).

The infusion of salbutamol was associated with a similar pattern of hemodynamics to those observed in dopexamine treated animals (Table 1, Figure 1, Supplementary Table 1, Supplementary Figure 2), and there was also no improvement in indices of tissue perfusion (Supplementary Figure 3). Compared to controls, salbutamol significantly reduced leucocyte-endothelial adhesion (S: 365 ± 49 mm^−2^, *p* < 0.01 vs. control) (Figure 2). Salbutamol also appeared to prevent arteriolar vasoconstriction, but unlike dopexamine was not associated with any change in FCD in any layer of the muscularis [Figure 2 (mid, right), Supplementary Figure 4] and had no effect on organ injury (Figure 3).

## Discussion

The principal findings of this experiment were that in a rodent model of laparotomy and endotoxemia, clinically relevant doses of dopexamine were associated with decreased leucocyte-endothelial adhesion and reduced arteriolar constriction in the intestinal microvasculature. However, with the exception of an increase in heart rate, dopexamine infusion was not associated with any systemic cardiovascular effects and in particular, relative cardiac index was not improved. In the higher dose dopexamine group an amelioration of renal dysfunction as assessed by plasma creatinine was observed. Almost all these findings were replicated by the β_2_-adrenoceptor agonist salbutamol though salbutamol failed to improve renal function (28).

This study provides evidence that dopexamine modulates the inflammatory response by reducing leucocyte-endothelial adhesion. We have previously demonstrated that in addition to reducing the pro-inflammatory cytokine response, dopexamine may also reduce the expression of leucocyte surface integrins following endotoxemia. We have also previously demonstrated a reduction in neutrophil infiltration in the lung of dopexamine treated endotoxaemic rats (28). It is possible that these observations are linked, such that during endotoxemia, a dopexamine mediated reduction in surface integrin expression results in reduced leucocyte-endothelial adhesion and consequential neutrophil transmigration into the tissues. Although this experiment does not elucidate the cellular events that result in reduced leucocyte-endothelial adhesion, the similar effects of salbutamol suggest a β_2_-adrenoceptor mediated mechanism. In this regard it has been shown that β_2_- and non-β_2_-adrenoceptor mediated elevations of cAMP reduce leucocyte adhesion (31, 32), whilst tonic activity of Protein Kinase A prevents β_2_-integrin activation (33). Other factors including, but not limited to, an amelioration of pro-inflammatory cytokines or drug effects on vascular endothelium may be of equal or greater relevance to these observed changes. Furthermore, the relevance of reduced leucocyte-endothelial adhesion to the effect of dopexamine on organ function is also unclear because although salbutamol reduced leucocyte adhesion in post-capillary venules it did not ameliorate renal injury.

The reduction in arteriolar vasoconstriction by dopexamine is consistent with the preservation of arteriolar diameters in villus arterioles and hepatic sinusoids observed in previous endotoxemia studies with dopexamine. In those studies, dopexamine treated endotoxaemic animals had an associated preservation of total organ and microvascular blood flow compared to untreated groups (23, 26). However, in our previous study we observed a reduction in ileal red cell flux during endotoxemia (28). Assuming the same effect occurred in this study, then the similar muscularis FCD in control and sham animals suggests that any perfusion defect (assessed by FCD) must occur in the mucosa, as has been shown in other studies using intravital microscopy (34). The non-significant trend to reduction in longitudinal FCD in dopexamine treated animals may then indicate a dopexamine mediated re-distribution of blood from the outer layer of the ileum to the hypoxia prone mucosa. In this regard it is interesting to note that although salbutamol and dopexamine had similar effects on arteriolar diameters, salbutamol did not show any tendency to produce this effect on longitudinal layer FCD, did not improve renal function and showed no tendency to a reduced plasma lactate either. These differences, despite the similarity of effects otherwise, are notable and warrant further study.

Previous clinical studies in major surgery including those by our own group have emphasized the role of cardiovascular optimisation in enhancing tissue oxygen delivery to reduce peri-operative morbidity (7, 8). Early studies drove clinicians to suggest this approach was beneficial as it reduced potentially harmful tissue ischaemia (35). The importance of maintaining tissue perfusion is still supported by modern studies from peri-operative medicine where MAP is clamped at higher levels and shown to reduce the incidence of post-operative morbidity (36). This may also relate to improvements in microvascular perfusion (37). However, we previously showed in surgical patients kept to a narrow MAP range that peri-operative stroke-volume guided fluid management protocols with continuous 0.5 μg kg^−1^ min^−1^ dopexamine infusion produced improvements in tissue oxygenation but without beneficial effects on markers of systemic inflammation or organ dysfunction (8). We also showed in a rodent model that dopexamine 1–2 μg kg^−1^ min^−1^ brings about potent immunomodulatory effects that are associated with improved organ function despite MAP and microvascular flow being similar to controls (28). This suggests that under surgical conditions therapeutic benefit is achievable through modulation of the host response to tissue injury and that further increases in tissue oxygenation or blood flow when perfusion is already guaranteed are redundant. In support of this, an analysis of surgical trials shows that patients with higher levels of baseline systemic inflammation are more likely to develop surgical complications (38). Similarly patients with impaired pre-operative microvascular function (who are known to have higher levels of inflammatory markers) are also more likely to suffer later complications (37, 39). Considering that previous studies have shown that surgical stress is associated with an upregulation of chemokines in the peritoneum and lungs, modulation of the host response to surgery becomes a potential explanation for the beneficial effects of dopexamine (40). However, the failure of salbutamol to improve renal function while producing a very similar spectrum of immune effects to dopexamine suggests that the mechanisms of renal protection with dopexamine are not necessarily only related to β_2_-adrenoceptor mediated effects on leucocyte-endothelial adhesion.

Differential abilities of dopexamine and salbutamol to increase cellular cAMP may explain divergent effects on renal function (41). This might be the case if dopaminergic receptor activation by dopexamine further increased cAMP levels above that provided by β_2_-adrenoceptor activation (5). The importance of increasing cAMP is that regulated cell death that causes tissue injury in acute kidney injury is inhibited by cAMP mediated-pathways, as is mitochondrial biogenesis which is required for enhanced recovery from cell stress (42, 43). On the other hand β_2_-adrenoceptor activation has also been shown to alter systemic metabolism to increase tissue tolerance of injury (44). Therefore, unexpected differential effects of the drugs at β_2_-adrenoceptors or in cAMP generation provide two mechanisms through which tissue damage can be minimized during the hours during and after emergency laparotomy and elective major abdominal surgery. This might also provide some explanation for the opposite findings in trials of β_2_-adrenoceptor agonism in acute respiratory distress syndrome (45). In those trials a week long infusion of higher doses of salbutamol were used to try and improve outcomes through a reduction in extravascular lung water but resulted in increased mortality. On the other hand in peri-operative medicine shorter-term infusions of similar agents are used to try and minimize tissue damage and organ dysfunction that can result from major surgery—trials in this setting with dopexamine have not shown any signal to increased mortality (7).

Several findings of this study are consistent with previous investigations. The haemodynamic effects of endotoxemia with or without dopexamine were replicated here and are in keeping with other studies (20, 28). Findings of intestinal arteriolar constriction are in keeping with the intense splanchnic vasoconstriction and rapid reduction of blood flow seen following endotoxemia and shock in rodents (20, 28, 46). This study, in keeping with other studies, found arteriolar constriction could be ameliorated by dopexamine (23). Similar studies have found an increase in adherent leucocyte numbers in intestinal or mesenteric post-capillary venules that could be ameliorated by dopexamine (19, 20). A significant increase in adherent leucocytes (reduced by dopexamine) and a decrease in rolling leucocyte numbers in post-capillary venules at two and a half hours would likely have resulted in leukopenia, as found in other studies (19–23). However, some findings of the experiment reported here, such as the failure of endotoxemia to decrease longitudinal and circular muscularis functional capillary density are not consistent with previous studies (20). These inconsistencies are likely to be the result of differences in the endotoxin serotype, dose, method of administration and fluid loading conditions of each experiment. Our study also appears to contradict the findings of Schmidt et al. regarding the role of β_2_-adrenoceptor agonism in leucocyte-endothelial adhesion (19). Importantly, our study design avoided the ablation of both endogenous and exogenous β_2_–adrenoceptor agonism that may account for differences in findings between the two studies.

Our study has several strengths. The use of IVM gave qualitative and quantitative data that is unobtainable from laser Doppler flowmetry studies (20). The nature of endotoxemia was modified, using peptidoglycan, which increases the generalizability of these findings outside of Gram negative septicaemia alone. Although the duration and nature of endotoxemia differed from our previous study, the model behaved in a similar fashion to our previous study with respect to hemodynamics, markers of perfusion and resultant organ dysfunction. With respect to biochemical markers of tissue perfusion, it is possible that the lack of statistical significance in the D1 group where lactic acidosis and base deficit were less severe (as in our previous study) is the result of smaller sample sizes. If correct, it is notable that salbutamol neither showed any signal to an amelioration of plasma lactate nor resulted in any amelioration of renal dysfunction. This could suggest there are additional important mechanisms of action of reducing organ injury that dopexamine possesses (as discussed above).

There are also limitations to the study performed. Although there were many similarities with our previous experiments, fundamental differences in design mean that it is not possible to be certain that the models behaved in an identical fashion. Secondly, although the use of IVM permitted direct visualization of the intestinal microcirculation, expected differences in FCD were not seen between shams and controls. This may have related to the mild severity of the model (note no significant hepatic dysfunction was observed in any group), to visualizing the intestinal microcirculation too early in the course of the experiment or even to the volume of fluid administered. In this regard the inability to observe changes in the microvascular bed over the entire course of the experiment was a weakness of this study. Furthermore, although arteriolar diameters and muscularis FCD were observed, the inability to measure center-line red cell velocity, mucosal FCD and mucosal inflow arteriolar diameters prevents a complete picture of the distribution of intestinal blood flow being made. Although reductions in leucocyte-endothelial adhesion were observed, it is not possible to ascertain the relative importance of this phenomenon to the reduction in organ injury seen in this or previous experiments. Derivation of the surrogates relative stroke volume and relative cardiac index from infra-renal blood flow is recognized in the literature. Nevertheless, it should be noted that cardiac index may have differed between groups and the effects of different doses of vasoactive drugs may have led to differing organ blood flows above the level of measurement, none of which could have been detected by an infra-renal flow probe. Regarding the use of dopexamine and salbutamol, dose equivalence was based on previous studies in isolated tracheal preparations (30). Although hemodynamics were similar between D1 and S groups suggesting the dose selection was probably correct, it is still not possible to be certain that the effect at the β_2_-adrenoceptor was identical for both drugs. This may be compounded by the fact that salbutamol is a mix of two enantiomers, adding further complexity to the comparison. In this regard the use of a selective β_2_-adrenoceptor antagonist to further disentangle the role of this drug effects will be useful in future studies. Finally, although the use of peptidoglycan increases the generalizability of these findings outside of Gram negative septicaemia, the choice of an endotoxin based model may still be criticized for lack of a true clinical correlate.

In summary, we present experimental evidence confirming that clinically relevant doses of dopexamine reduce leucocyte-endothelial adhesion in the intestinal microvasculature and are associated with improved renal function at clinically relevant doses. As a consequence of our experiments some avenues warrant further research. The relationship between β_2_-adrenoceptor signaling and downstream effects on leucocyte CD11b expression, tissue tolerance mechanisms and inhibition of regulated cell death deserve further attention. The effect of dopexamine on microvascular recruitment and its relationship to cardiac index under differing fluid regimes and also the effect of dopexamine on the distribution of microvascular blood flows are two areas that also warrant further study given the disparity seen in results of our studies and others (8, 20, 23). Although peri-operative dopexamine use has been shown to be safe in randomized controlled trials and a Bayesian analysis of the OPTIMIZE trial suggested a high probability of superiority of treatment efficacy, a new randomized controlled trial of peri-operative optimisation using β_2_-adrenoceptor agonists including dopexamine is underway and will inform clinicians definitively regarding the role of peri-operative dopexamine and haemodynamic optimisation (7, 47, 48).

## Data Availability Statement

The datasets generated for this study are available on request to the corresponding author.

## Ethics Statement

The protocol was approved on 17/10/2011 under the project license number PPL 70/6526, by the AWERB (Animal Welfare and Ethical Review Body of Queen Mary University of London). All procedures were performed in accordance with the United Kingdom Home Office Guidance on the Operation of the Animals (Scientific Procedures) Act 1986.

## Author Contributions

MB carried out the *in vivo* studies and statistical analysis of all data. NP provided advice during *in vivo* studies and on study design. TA took offline measurements from intravital microscopy videos. CT participated in the design and coordination of the study and provided guidance throughout. RP conceived the study, participated in its design and co-ordination, and helped with statistical analysis. RP, CH, CT, and MB together drafted the manuscript. All authors read and approved the final manuscript.

## Conflict of Interest

RP has received equipment loans from LiDCO Ltd and has performed consultancy work for Edwards Lifesciences and Massimo Inc. RP is a member of the associate editorial board of the British Journal of Anaesthesia. CT is CEO of William Harvey Research Limited, which is a CRO and has conducted contracted research in the area of critical care. CT is also Senior Associate Editor for the journal Shock. The remaining authors declare that the research was conducted in the absence of any commercial or financial relationships that could be construed as a potential conflict of interest.
