# Supplementary material for: The Effect of β2-Adrenoceptor Agonists on Leucocyte-Endothelial Adhesion in a Rodent Model of Laparotomy and Endotoxemia
Source: Front Immunol. 2020 May 21;11:1001. doi: 10.3389/fimmu.2020.01001 (PMC7326121; doi:10.3389/fimmu.2020.01001)
Supplement: Supplementary file 1 [file Data_Sheet_1.PDF]

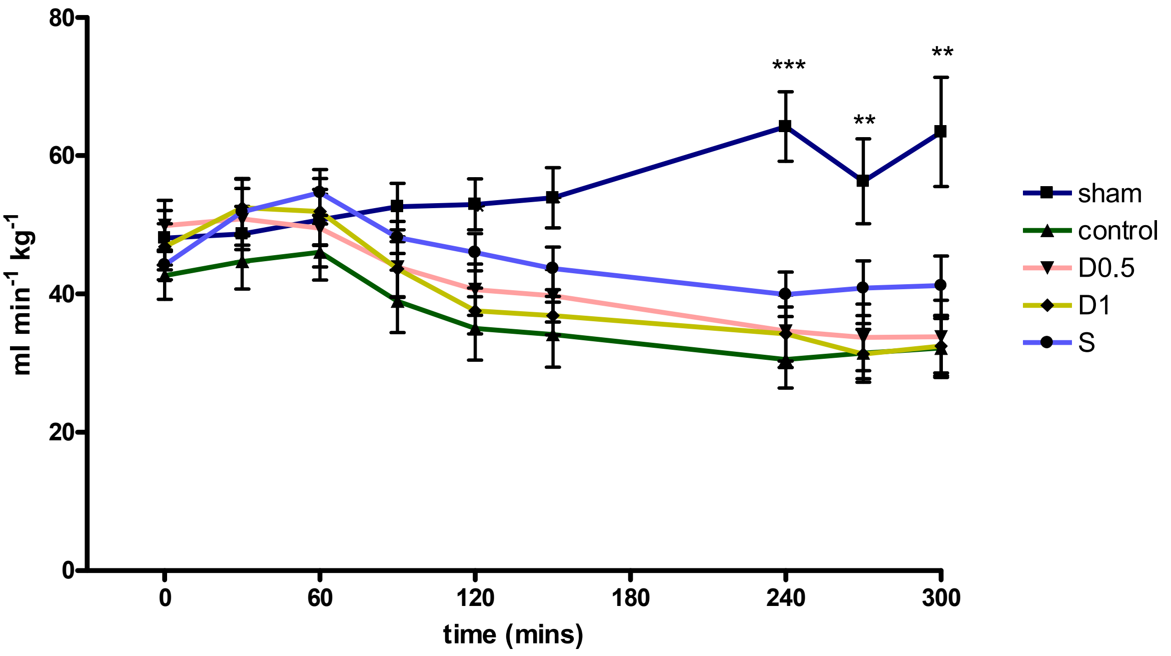

Supplementary Figure 1 Relative cardiac index during 5 hours of laparotomy and endotoxaemia recorded every 30 minutes. Values not plotted for t180 – t210 (animals were undergoing IVM at this time and aortic flow could not be measured).
Relative cardiac index in controls differed significantly for at least the last 60 minutes of the experiment when compared to shams. However no significant differences were observed between controls and animals treated with dopexamine or salbutamol at any time. The mean change in relative cardiac index from baseline to end experiment was significant in only control and dopexamine treated groups (also see Table 1). Data presented as mean (SEM). One-way ANOVA at each time point (Bonferroni's post-tests, *$p<0.05$, **$p<0.01$, ***$p<0.001$ sham compared with controls).

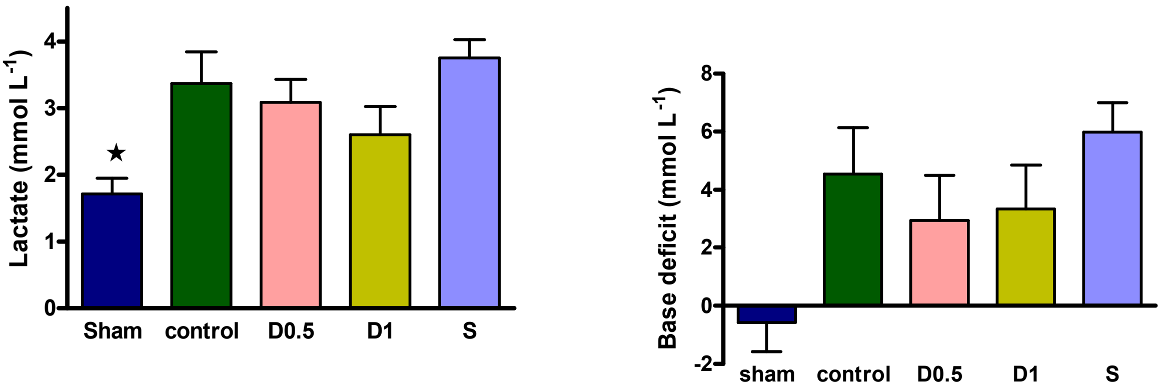

Supplementary Figure 2 Plasma lactate (n=6 all groups) and base deficit (all n=6 except control and D0.5, n=5 each) sampled 5 hours after laparotomy and endotoxaemia: In control animals, lactate significantly increased compared with shams. A similar observation was made for base deficit (see below). Data presented as mean (SEM). Lactate: One-way ANOVA (Bonferroni's post-tests, *$p<0.05$ vs. control). Base deficit: One-way ANOVA $p = 0.0206$ (no groups positive in post-test, although $p<0.05$ when comparing sham and control with unpaired t-test).

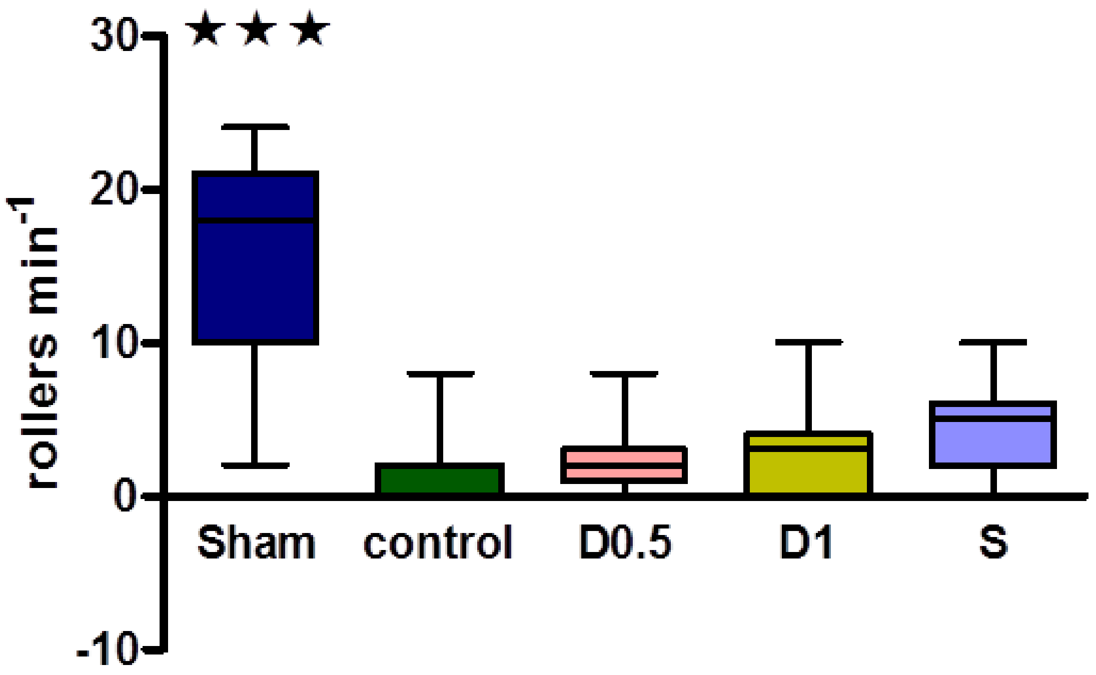

Supplementary Figure 3 Numbers of rolling leucocytes in ileal post-capillary venules after 2.5 hours of laparotomy and endotoxaemia (n=6 all groups). Numbers of vessels observed per group ranged from 8-18. In contrast to control animals, a significantly greater degree of venular rolling was observed in shams. Data presented as median (IQR, range). Kruskal Wallis test (Mann-Whitney post-tests with Bonferroni correction, ***p<0.001 compared to controls).

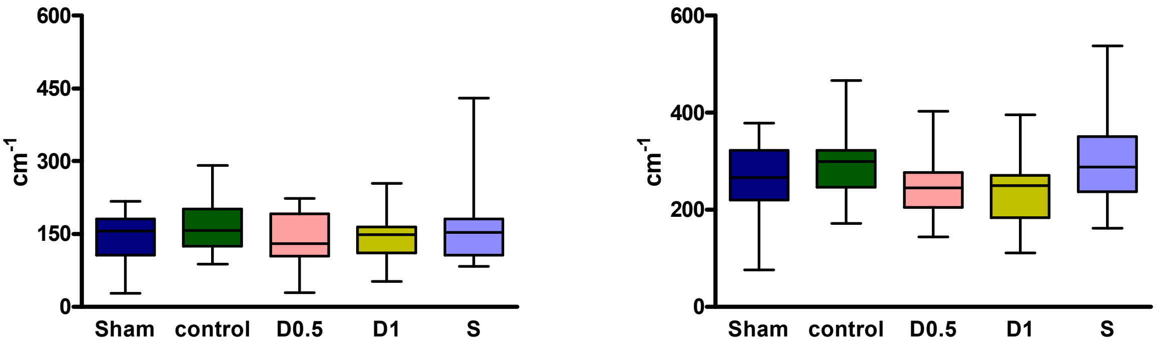

Supplementary Figure 4 Intestinal functional capillary density in circular layers (left) and both circular and longitudinal layers combined (right) of the ileal muscularis (n=6 all groups) after 2.5 hours of laparotomy and endotoxaemia. Number of images per group ranged from 16-25. No significant differences were found between groups for FCD in the circular layer of the muscularis. The total FCD in the ileal muscularis (circular plus longitudinal) also fell outside of the limits of significance (p=0.058). Data presented as median (IQR, range).
