# Supplementary material for: The Effect of β2-Adrenoceptor Agonists on Leucocyte-Endothelial Adhesion in a Rodent Model of Laparotomy and Endotoxemia
Source: Front Immunol. 2020 May 21;11:1001. doi: 10.3389/fimmu.2020.01001 (PMC7326121; doi:10.3389/fimmu.2020.01001)
Supplement: Supplementary file 2 [file Table_1.DOCX]

**Supplementary Table 1** Baseline characteristics, arterial blood gas results and temporal changes in haemodynamics for all groups (n=6 all groups). Data presented as mean (SEM) when all groups normally distributed, otherwise median (IQR) if ≥ 1 group not normally distributed. Weight data was not normally distributed for all groups but both IQR and SEM are shown, respectively. Paired t-tests of baseline vs end experiment for mean changes (*p<0.05, **p<0.01).

|  | ***Sham*** | ***Control*** | ***D 0.5*** | ***D1*** | ***S*** |
| --- | --- | --- | --- | --- | --- |
| *Weight (g)* | 295  (250 – 318, 13) | 305  (290 – 320, 6) | 310  (290 – 330, 9) | 320  (300 – 335, 7) | 300  (290 – 305, 3) |
| *Thiopental*  *(mg kg^-1^)* | 186.6 (8.8)** | 141.4 (5.0) | 141.6 (6.4) | 142.2 (7.0) | 157.5 (7.3) |
| *Baseline haematocrit (%)* | 39.0  (35.5 – 42.5) | 42.5  (38.0 – 43.5) | 45.5  (39.0 – 49.0) | 42.0  (41.5 – 43.5) | 40.5  (36.0 – 45.5) |
| *End experiment haematocrit (%)* | 42.5 (1.4) | 41.0 (1.0) | 42.8 (0.6) | 39.3 (1.0) | 37.5 (2.2) |
| *End experiment pH* | 7.38 (0.02) | 7.39 (0.02) | 7.36 (0.01) | 7.39 (0.02) | 7.34 (0.02) |
| *End experiment P_a_CO_2_ (kPa)* | 5.9 (0.4)* | 4.3 (0.4) | 4.9 (0.3) | 4.7 (0.4) | 4.7 (0.1) |
| *End experiment P_a_O_2_ (kPa)* | 11.1  (10.0 – 12.0) | 12.7  (11.7 – 13.6) | 11.6  (10.2 – 12.6) | 10.2  (9.9 – 13.8) | 11.1  (9.9 – 12.3) |
| *Mean change in HR during experiment*  *(bpm)* | -14  (11) | 49  (10)** | 57  (11)** | 87  (18)** | 54  (9) ** |
| *Mean change in MAP during experiment*  *(mmHg)* | 6 (10) | -23 (9) (P=0.052) | -27 (6)* | -15 (6) | -26 (8)* |
